# Predicting expected progeny difference for marbling score in Angus cattle using artificial neural networks and Bayesian regression models

**DOI:** 10.1186/1297-9686-45-34

**Published:** 2013-09-11

**Authors:** Hayrettin Okut, Xiao-Liao Wu, Guilherme JM Rosa, Stewart Bauck, Brent W Woodward, Robert D Schnabel, Jeremy F Taylor, Daniel Gianola

**Affiliations:** 1 Department of Animal Sciences, University of Wisconsin, Madison, WI 53706, USA; 2 Department of Animal Science, Biometry and Genetics Branch, University of Yuzuncu Yil, Van 65080, Turkey; 3 Department of Dairy Science, University of Wisconsin, Madison, WI 53706, USA; 4 Department of Biostatistics and Medical Informatics, University of Wisconsin, Madison, WI 53706, USA; 5 GeneSeek, a Neogen Company, Lincoln, NE 68521, USA; 6 NextGen Consulting, Atlanta, GA, USA; 7 Division of Animal Sciences, University of Missouri, Columbia, MO 65211, USA

## Abstract

**Background:**

Artificial neural networks (ANN) mimic the function of the human brain and are capable of performing massively parallel computations for data processing and knowledge representation. ANN can capture nonlinear relationships between predictors and responses and can adaptively learn complex functional forms, in particular, for situations where conventional regression models are ineffective. In a previous study, ANN with Bayesian regularization outperformed a benchmark linear model when predicting milk yield in dairy cattle or grain yield of wheat. Although breeding values rely on the assumption of additive inheritance, the predictive capabilities of ANN are of interest from the perspective of their potential to increase the accuracy of prediction of molecular breeding values used for genomic selection. This motivated the present study, in which the aim was to investigate the accuracy of ANN when predicting the expected progeny difference (EPD) of marbling score in Angus cattle. Various ANN architectures were explored, which involved two training algorithms, two types of activation functions, and from 1 to 4 neurons in hidden layers. For comparison, BayesCπ models were used to select a subset of optimal markers (referred to as feature selection), under the assumption of additive inheritance, and then the marker effects were estimated using BayesCπ with π set equal to zero. This procedure is referred to as BayesCpC and was implemented on a high-throughput computing cluster.

**Results:**

The ANN with Bayesian regularization method performed equally well for prediction of EPD as BayesCpC, based on prediction accuracy and sum of squared errors. With the 3K-SNP panel, for example, prediction accuracy was 0.776 using BayesCpC, and ranged from 0.776 to 0.807 using BRANN. With the selected 700-SNP panel, prediction accuracy was 0.863 for BayesCpC and ranged from 0.842 to 0.858 for BRANN. However, prediction accuracy for the ANN with scaled conjugate gradient back-propagation was lower, ranging from 0.653 to 0.689 with the 3K-SNP panel, and from 0.743 to 0.793 with the selected 700-SNP panel.

**Conclusions:**

ANN with Bayesian regularization performed as well as linear Bayesian regression models in predicting additive genetic values, supporting the idea that ANN are useful as universal approximators of functions of interest in breeding contexts.

## Background

The availability of genome-wide dense marker panels for many species of plants and animals has opened doors for incorporating genomic information into practical breeding programs, an approach known as genomic selection [1]. It is now easy to generate genome-wide scans with more than one million SNPs (single nucleotide polymorphisms) but these huge databases pose challenges in computational capacity, data analysis and interpretation of results for genomic selection [2]. For example, even with an initial screening to reduce the number of markers to less than ten thousand SNPs, it is still not feasible for most computational platforms to evaluate all combinations of SNP associations, even when low-dimension interactions are explored [3]. Hence, reduction of dimensionality and feature extraction arguably play pivotal roles in current genomic studies [4]. The intensive computation inherent in these problems has altered the course of methodological developments, and the same is true for genomic selection [5].

In the genome-enabled prediction of genetic merit for breeding purposes, parametric statistical methods tend to make strong assumptions about functional forms and the statistical distribution of marker effects. On the one hand, ridge regression best linear unbiased prediction assumes that all markers have an effect on the trait of interest, and that these effects share a common variance in their distribution. This simple assumption is obviously not true in reality. On the other hand, hierarchical linear Bayesian regression models, such as BayesA and BayesB [1], allow marker effects to be estimated with differential shrinkage. However, posterior inference, in particular for the variances of marker effects, depends heavily on the prior assumptions used in these models [6]. Hence, BayesCπ [7] was proposed to overcome some of the above mentioned drawbacks. A BayesCπ model postulates an unknown probability π that a SNP has no effect at all on the trait, while all non-zero SNP effects are assumed to be random samples from a normal distribution with null mean and a common variance. Recently, there has been interest in the use of non-parametric methods for the prediction of quantitative traits, such as reproducing kernel Hilbert space regressions [8,9], radial basis function models [10] and non-parametric Bayesian models with Dirichlet process priors [11]. These nonparametric methods make weaker assumptions and can be more flexible for describing complex relationships [12].

Artificial neural networks (ANN), also known as neuro-computational models, provide an appealing alternative for genome-enabled prediction of quantitative traits [13,14]. These machine learning methods can act as universal approximators of complex functions [15] because they are capable of capturing nonlinear relationships between predictors and responses and can adaptively learn complex functional forms, through a series of transformations (i.e., activation functions) driven by parameters. Multilayer feed-forward is the most common architecture used in ANN, which consists of neurons assembled into layers. The first layer is called the input layer (the left-most layer of the ANN) that accepts data (e.g., SNP genotypes) from sources external to the network, and the last layer (the right-most layer) is called the output layer that contains output units of the network. In between these two layers are so-called hidden layers, because their values are not observed in the training set. Hence, an ANN architecture is specified by the number of layers, the number of neurons in each layer, and the type of activation functions that are used. Neurons in each layer are connected to the neurons from the previous and the subsequent layer through adaptable synaptic weights.

The network weights are evaluated in two steps: the feed-forward and the back-propagation steps. In the feed-forward step, information comes from the input layer and each unit evaluates its activation function, which is transmitted to the units connected to the output layer. The back-propagation step consists of running the whole network backwards to minimize the error function in the space of weights using the method of gradient descent. A set of weights that minimizes the error function is considered to be a solution of the learning problem for the ANN.

Determination of the number of neurons to be placed in the hidden layer is a critical task in the design of ANN. A network with an insufficient number of neurons may not be capable of capturing complex patterns. In contrast, a network with too many neurons will suffer from over-parameterization, leading to over-fitting and poor predictive ability of yet to be observed data. Two popular techniques to overcome the over-fitting problem in ANN models are Bayesian regularization and cross-validated early stopping (CVES). Bayesian regularization (BR) constrains (shrinks) the magnitude of the networks weights, improves generalization and allows bias in parameter estimates towards values that are considered to be plausible, while reducing their variance; thus, there is a bias-variance trade-off [13]. Unlike other back-propagation training algorithm methods that use a single set of parameters, BR considers all possible values of network parameters weighted by the probability of each set of parameters. In practice, Bayesian regularized ANN can be computationally more robust and cost-effective than standard back-propagation nets because they can reduce or eliminate the need for lengthy cross-validation.

With cross-validated early stopping (CVES) methods, the training data set is split into a new training and a validation set and a gradient descent algorithm is applied to the new training data set. The ANN performs an iterative process, first using the training data set to calculate the weight and bias estimates, and then applies these parameter estimates in the validation data set to calculate the prediction errors. The process iterates repeatedly, substituting parameter estimates from the training data set into the validation data set to find the smallest possible average prediction errors for the validation data set. Training ceases when the error in the validation data set increases in certain consecutive epochs (iterations) in order to avoid the problem of over-fitting (the number of consecutive epochs is 6 by default in MATLAB). The ANN parameter estimates with the best performance in the validation set is then used on the testing data to evaluate the predictive ability of the network.

In a previous study, ANN with BR outperformed a benchmark linear model when predicting milk yield in dairy cattle or grain yield of wheat [14]. However, because breeding values are defined in terms of linear functions based upon additive inheritance, the predictive performance of ANN relative to linear additive systems is of some interest. This motivated the present study, in which the aim was to investigate the accuracy of ANN for predicting expected progeny differences (EPD) for marbling score in Angus cattle relative to hierarchical linear Bayesian regression models. Various ANN architectures were explored, which involved two training algorithms, two types of activation functions, and from 1 to 4 neurons in hidden layers.

## Methods

### Data sets

**Figure 1:**
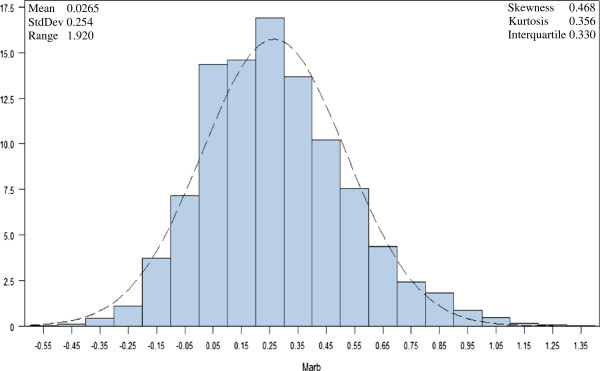
Histogram and density plot of deregressed expected progeny differences for marbling score for 3079 Angus cattle.

The data contained 3079 registered Angus bulls, genotyped with the Illumina BovineSNP50 BeadChip, from which the SNP content for the Illumina Bovine3K BeadChip [16] was extracted. After data quality control and screening, a total of 2421 polymorphic SNPs were retained for analysis. The target variable to be predicted was EPD for marbling score, which had been computed by the American Angus Association using BLUP (best linear unbiased prediction) for each of these animals, based upon their pedigree data and progeny carcass and ultrasound data [17]. In animal breeding, an EPD is defined as the predicted performance of a future offspring of an animal for a particular trait (marbling score), calculated from measurement(s) of the animal’s own performance and/or the performance of its relatives under the assumption of additive inheritance. Hence, EPD represent a typical linear system since the EPD of an individual can be represented as a linear function of the EPD of relatives and a residual term that reflects the fact that an individual inherits a random sample of the genes present within its parents. The distribution of marbling score EPD in the Angus sample was symmetric and suggested a normal distribution (Figure 1), with mean and standard deviation estimated at 0.0265 and 0.254, respectively [18].

### Statistical methods

#### Artificial neural networks

**Figure 2:**
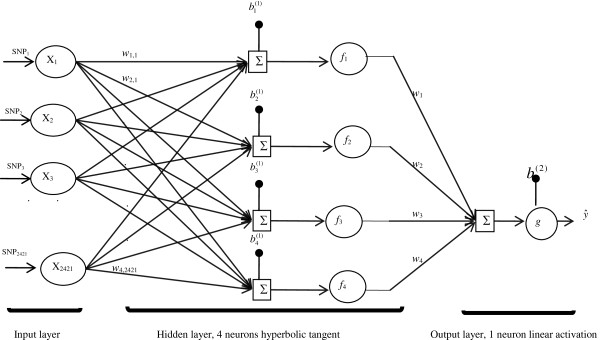
**Schematic representation of a three-layer feed-forward neural network.** Genotypes for 2421 (or 700) SNPs were used as inputs **x**_*j*_ = {*x*_*ij*_|*i* = 1, 2, …, *n*}, where *n* is the number of individuals with genotypes; each SNP was connected to up to 4 neurons via coefficients *w*_*kj*_, where *k* denotes neuron and *j* denotes SNP; here, *w*_*k*_ is a weight from a hidden layer units to the output unit, *f*_*k*_ is an activation function applied to hidden layer units (e.g., the hyperbolic tangent), *g* is an activation function applied to the output layer unit (e.g., linear), *b*^(1)^ and *b*^2^ are biases of hidden and output layer units, and $\hat{y}$ is a predicted value.

A schematic presentation of a multilayer perceptron (MLP) feed-forward of an ANN is presented in Figure 2. This is a multi-layer feed-forward neural network with a single middle (hidden) layer and four neurons. Training an ANN involves the minimization of an error function that depends on the network’s synaptic weights (the w’s in Figure 2), and these weights are iteratively updated by a learning algorithm, to approximate the target variable. The updating is usually accomplished by back-propagating the error, which is essentially a non-linear least-squares problem [19]. Back-propagation is a supervised learning algorithm based on a suitable error function, the values of which are determined by the target (i.e., marbling EPD) and the mapped (predicted) outputs of the network (i.e., fitted values of marbling EPD). Weights in an MLP architecture are determined by a back-propagation algorithm to minimize the sum of squares of errors using gradient-descent methods [13]. During the training, weights and biases in the ANN are successively adjusted based on the input and the desired output. Each iteration of feed-forward in a MLP constitutes two sweeps: forward activation to produce a desired output, and backward propagation of the computed error to update the values for the weights and biases. The forward and backward sweeps are repeatedly performed until the ANN solution agrees with the desired value to within a pre-specified tolerance [20,21].

Let $x_{j}^{'}=\left(x_{1j},x_{2j}......,x_{\mathrm{nj}}\right)$ be a row vector that contains SNP genotypes for all *i* = 1,…,n individuals, for the *j*^th^ SNP, where *j* = 1,…..,*p*, and *p* = 700 (referred to as the 700-SNP panel) or *p* = 2421 (referred to as the 3K-SNP panel). In an ANN, SNP genotypes are connected to each neuron in a single hidden layer via weights *w*_*kj*_*,* for *k* = 1, …, *K* neurons, with each weight defining a specific SNP-neuron connection with appropriate biases (intercepts), $b_{1}^{\left(1\right)},b_{2}^{\left(1\right)},......,b_{k}^{\left(1\right)}$, each pertaining to a specific neuron. The input into neuron *k*, prior to activation is expressed linearly as $b_{k}^{\left(1\right)}+\sum_{j=1}^{p}w_{\mathrm{kj}}x_{j}$, where *p* = 700 or 2421, and $b_{k}^{\left(1\right)}$ is the bias parameter defined in the hidden layer and this quantity is then transformed using some linear or nonlinear activation function (*f*_*k*_) as:

(1) $$f_{k}\left(b_{k}^{\left(1\right)}+\sum_{j=1}^{p}w_{\mathrm{kj}}x_{j}\right).$$

The above is the output of the hidden layer, which in turn is delivered to the output layer (e.g., each neuron *k* in the hidden layer sums ANN input *x*_*j*_ after multiplying them by the strengths of the respective connection weights, *w*_*kj*_, and adds biases *b*_*k*_ and then computes its output as a function of the sum), and is collected as:

(2) $$b^{\left(2\right)}+\sum_{k=1}^{K}w_{k}f_{k}\left(b^{\left(1\right)}+\sum_{j=1}^{p}w_{\mathrm{ij}}x_{j}\right),$$

where *w*_*k*_ is the weight specific to the *k*^th^ neuron (*k* = 1, 2, …, *K*)*,* and *b*^(2)^ is the bias parameter defined in the output layer. Next, quantity (2) is activated with the following function:

(3) $$g\left(.\right)=g\left[\sum_{k=1}^{K}w_{k}f_{k}\left(.\right)+b^{\left(2\right)}\right].$$

The above yields the fitted marbling EPD, ${\hat{y}}_{i}$, for each individual in the training set. Finally, the predicted value of marbling score EPD can be computed in the testing set using a formula similar to (3). We used the hyperbolic tangent sigmoid and linear (identity) activation functions in the hidden and output layers, respectively.

We used BR and scaled conjugate gradient (SCG) back-propagation as training algorithms. The basic idea of back-propagation algorithms is to adjust weights in the steepest descent direction (negative of the gradient), such that the objective function decreases most rapidly [22]. However, in practice, this does not necessarily produce the fastest convergence, although the function may decrease most rapidly along the negative of the gradient. Several conjugate gradient algorithms have been proposed, in which a search is performed along conjugate directions, generally leading to faster convergence to a local function minimum than steepest descent directions [22,23]. On the one hand, with the exception of SCG, the conjugate gradient algorithms use linear searches at each iteration and thus, are computationally expensive as they require that the network response to all training inputs be computed several times for each search. On the other hand, the SCG algorithm combines a model-trust region approach with the conjugate gradient approach and uses a step size scaling mechanism to avoid time-consuming linear searches [23]. Hence, SCG can significantly reduce the number of computations performed in each iteration, but may require more iterations to converge than do the other conjugate gradient algorithms [22]. SCG uses the CVES technique to prevent over-fitting, which is commonly used in neural networks and can improve their predictive ability (generalization).

Training proceeds as follows. Let the data set be *D* = {**y**, {**x**_*i*_}_*i* = 1,…,*n*_}, where **x**_*i*_ is a vector of inputs (SNP genotypes) for individual *i* and **y** is a vector of target variables (EPD). Once a set of weights, **w,** is assigned to the connections in the networks, this defines a mapping from the input **x**_*i*_ to the output ${\hat{y}}_{i}$. Let *M* denote a specific network architecture (in terms of numbers of neurons and choice of activation functions), then the typical objective function used for training a neural network using CVES is the sum of squared prediction errors (*E*_*D*_):

(4) $$E_{D}\left(D|w,M\right)=\sum_{i=1}^{n}{\left({\hat{y}}_{i}-y_{i}\right)}^{2}$$

for *n* input-target pairs defining *D*.

Regularization produces shrinkage of parameter estimates towards some plausible values, while at the same time reducing their variance. In Bayesian models, regularization techniques involve imposing certain prior distributions on the model parameters. In Bayesian ANN (e.g., BRANN), the objective function that was specified in (4) has an additional term that penalizes large weights, in the hope of achieving a smoother mapping. Gradient-based optimization is then used to minimize the following function, equal to a penalized log-likelihood,

(5) $$F=\beta E_{D}\left(\left(D\right|w,M\right)+\alpha E_{W}\left(\left(w\right|M\right),$$

where *E*_*W*_ (**w***|M*) is the sum of squares of network weights, *M* is the ANN architecture, and α and *β* are positive regularization parameters that must be estimated. The second term on the right hand side of (5), known as weight decay, favors small values of **w** and decreases the tendency of a model to over-fit the data. Hence, training involves a tradeoff between model complexity and goodness of fit. Large values of *α* lead to posterior densities of weights that are highly concentrated around zero, so that the weights effectively disappear, the model discounts connections in the network [24,25], and complex models are automatically self-penalized. From equation (5), if α << β, the fitting or training algorithm places the most weight on goodness of fit. If α >> β, emphasis is placed on reducing the magnitude of the weights at the expense of goodness of fit, while producing a smoother network response [26].

In an empirical Bayesian framework, the “optimal” weights are those that maximize the conditional posterior density *P*(**w**|*D*, *α*, *β*, *M*), which is equivalent to minimizing the regularized objective function *F* in equation (5). Minimization of *F* is identical to finding the (locally) maximum *a posteriori* estimates of **w,** denoted **w**^***MP***^, which minimize *E*_*D*_ using the back-propagation training algorithms [24]. However, this is possible only if *n* >*m*, where *m* is the number of parameters to be estimated.

Bayes theorem yields the posterior density of *α* and *β* as:

$$P\left(\left(\alpha ,\beta \right|D,M\right)=\frac{P\left(\left(D\right|\alpha ,\beta ,M\right)P\left(\left(\alpha ,\beta \right|M\right)}{P\left(\left(D\right|M\right)}.$$

If the prior density *P*(*α*, *β*|*M*) is uniform, maximization of *P*(*α*, *β*|*D*, *M*) with respect to *α* and *β* is equivalent to maximization of *P*(*D*|*α*, *β*, *M*). Bayesian optimization of the regularization parameters requires computation of the Hessian matrix of the objective function *F* evaluated at the optimum point **w**^***MP***^[27], but directly computing the Hessian matrix is not always necessary. As proposed by MacKay [28], the Gauss-Newton approximation to the Hessian matrix can be used if the Levenberg-Marquardt optimization algorithm is employed to locate the minimum of *F*[13,29,30]. The Levenberg–Marquardt training algorithm [31] achieves second-order training speed without computing the Hessian matrix. Briefly, the Hessian matrix is approximated as:

(7) H=J'J,

where **J** is the Jacobian matrix that contains first derivatives of the network errors with respect to network parameters (the weights and biases). The gradient is computed as:

(8) g=J'e,

and network parameters are updated as:

(9) $$w_{k+1}=w_{k}-\left(J'J+\mu I\right)\times J'e.$$

Here, *μ* is Levenberg's damping factor, which is adjusted at each iteration and guides the optimization process. If the reduction of error sum of squares is rapid, a smaller value of *μ* is used to bring the algorithm closer to the Gauss–Newton algorithm. Alternatively, the damping factor is increased to give a step to gradient descent direction if an iteration provides insufficient reduction in the error sum of squares [32]. Optimal values of regularization parameters, *α* and *β*, in BRANN can be calculated as:

(10) $$\alpha^{\mathrm{MP}}=\frac{\gamma }{2E_{w}\left(w^{\mathrm{MP}}\right)}$$

and

(11) $$\beta^{\mathrm{MP}}=\frac{n-\gamma }{2E_{D}\left(w^{\mathrm{MP}}\right)},$$

where 0 ≤ *γ* = *m* − 2*α*^*MP*^*tr*(**H**^***MP***^)^− 1^ ≤ *m* the number of effective parameters in the neural network, and *m* is the total number of parameters. Here **H**^**MP**^ is the maximum *a posteriori* estimate of H described in (7).

#### BayesCπ and BayesC with π = 0

Consider the following linear model:

(12) $$y_{i}=\mu +\sum_{j=1}^{p}x_{\mathrm{ij}}\alpha_{j}+e_{i},$$

where *y*_*i*_ is the marbling score EPD for the *i*^th^ animal; *μ* is the overall mean; *α*_*j*_ is the substitution effect associated with the *j*^th^ SNP (*j* = 1, …, *p*); *x*_*ij*_ is an indicator variable corresponding to the genotype at the *j*^th^ SNP (0, 1, 2) for the *i*^th^ individual, and $e_{i}\sim N\left(0,\sigma_{e}^{2}\right)$ is a residual term, where $\sigma_{e}^{2}$ is the residual variance.

The BayesCπ model [7] assumes that each SNP effect is null with probability *π*, or that it follows a normal distribution, $N\left(0,\sigma_{\alpha }^{2}\right)$, with probability 1- *π*, i.e.:

(13) $$\alpha_{i}|\pi ,\sigma_{\alpha }^{2}\left\{\begin{array}{ll} \sim N\left(0,\sigma_{\alpha }^{2}\right) & \mathrm{with}\;\mathrm{probability}\;\left(1-\pi \right)\\ =0 & \mathrm{with}\;\mathrm{probability}\;\pi \end{array}\right).$$

Here, $\sigma_{\alpha }^{2}$ is a variance common to all non-zero SNP effects, which is assigned a scaled inverse chi-square distribution, $\chi^{-2}\left(v_{\alpha },s_{\alpha }^{2}\right)$. Furthermore, the value of *π* is unknown and needs to be inferred, with the prior distribution of *π* taken to be uniform between 0 and 1,

(14) $$\pi \sim \mathrm{Uniform}\left(0,1\right).$$

A Bernoulli indicator variable, *δ*_*j*_, is introduced to facilitate sampling from the mixtures of the SNP effects, as follows:

$$p\left(\left(\delta_{i}\right|\pi \right)=\pi^{1-\delta_{i}}{\left(1-\pi \right)}^{\delta_{i}}.$$

Hence, unconditionally, the variable *α*_*j*_ follows a univariate-t distribution, $t\left(0,S_{\alpha }^{2},\upsilon_{\alpha }\right)$, if *δ*_*j*_ = 1, or is equal to zero otherwise [6]. Posterior inference of unknown parameters in the Bayesian model via Markov chain Monte Carlo (MCMC) implementation is described in [7]. With a subset of, say *k* ≤ *p*, selected markers, the statistical model takes the same form as (12), replacing *p* with *k* for the number of markers.

By assuming that all *k* of the selected SNPs (based on the posterior model probability and including the frequency of marker *k* during MCMC) have non-null effects on the quantitative trait, we define a BayesCπ model with *π* = 0, which was used for the statistical inference and model cross-validation subsequent to selection of markers (referred to as post-selection hereafter). So, posterior inference in BayesCπ with *π* = 0 was as for BayesC *π*, except that *π* was fixed at zero and hence sampling of the indicator vector **δ** was no longer relevant.

#### Computational implementation

MATLAB [31] was used to fit the ANN. Each neural network consisted of three layers (i.e., input, hidden and output layers). The number of neurons in a single hidden layer varied from 1 to 4. Each ANN had 2421 (or 700) inputs (SNPs). Before processing, MATLAB automatically rescaled all input and output variables using the “mapminmax” function such that they resided in the range [−1, +1], to enhance numerical stability. Two combinations of activation functions were used: (i) a set of hyperbolic tangent sigmoidal activation functions from the input layer to the hidden layer, plus a linear activation function from the hidden layer to the output layer, and (ii) a set of linear activation functions from the input layer to the hidden layer and from the hidden layer to the output layer. Training was stopped if any of the following criteria were met: (i) a maximum number (1000) of epochs was reached, (ii) performance had met a pre-specified (the performance function for feed-forward networks is the mean square error) level, (iii) the gradient was below a suitable target, or (iv) the Levenberg-Marquardt parameter exceeded 10^10^.

For each ANN architecture, eight replicates were run. Each replicate was independently initialized, in order to eliminate spurious effects caused by the starting values, and to improve predictive ability. The results were presented as averages across the eight replicates per ANN architecture.

BayesCπ with π set equal to zero (referred to as the BayesCpC procedure) was implemented via a high-throughput computing pipeline to select SNPs for post-selection statistical inference and cross-validation [5]. This pipeline ran multiple chains for both feature selection and cross-validation. A three-fold cross-validation approach was employed, in which the whole dataset was divided into three approximately equal portions, with two-thirds used for training and one-third used for testing, and the portions used for training and testing were rotated three times. Each cross-validation experiment was randomly replicated eight times. Three parallel MCMC chains were run for each feature-selection analysis, and each consisted of 50 000 iterations after a burn-in of 5000 iterations, thinned every tenth iterate. MCMC sampling for each cross-validation consisted of 100 000 iterations, with a burn-in of 10 000 iterations, thinned every tenth iterate.

## Results

### Determination of an optimal SNP panel size

The predictive performance of each ANN was examined using either the 3K-SNP panel or an optimal subset of 700 selected SNPs. The latter were derived from the 3K-panel, selected using the BayesCpC procedure with three-fold cross-validation. This was accomplished by examining the prediction performance of varying panel sizes from 100 to 2400 SNPs in 100-SNP increments, and the optimal set that gave the best prediction in cross-validations was identified. The reason for not choosing the optimal subset based on ANN models was because the selection tasks with a grid of 24 candidate SNP-panels of varying sizes were too computationally intensive for BRANN. Nevertheless, the parallel-BayesCpC pipeline handled this task easily, because all jobs were submitted to run in parallel on a cluster of dedicated computers.

**Figure 3:**
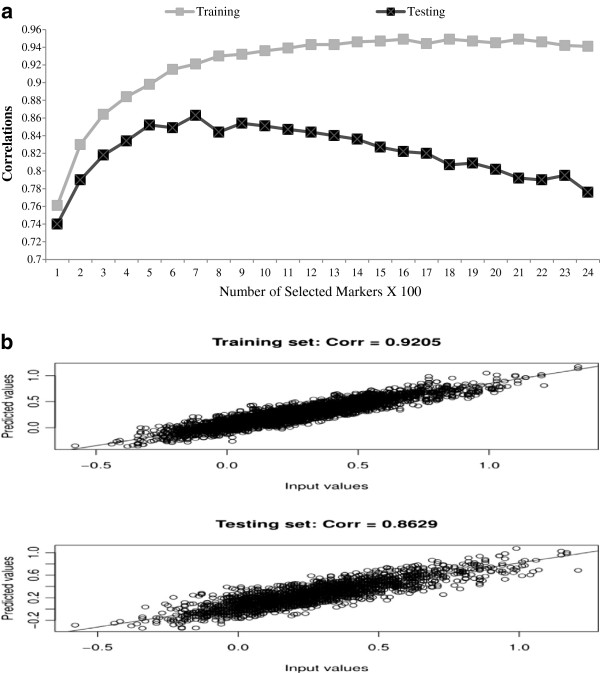
**Selection of an optimal SNP panel size for predicting marbling score expected progeny difference (EPD) using the Bayes CpC approach. (a)** Correlations between marbling score EPD and their fitted (predicted) values in the training and testing sets, where the SNP panel consisted of 100, 200,…, 2400 markers, respectively; **(b)** correlation between EPD (input values) and fitted values in the training set (upper) and correlation between EPD (input values) and predicted values in the testing set for the optimal set of 700 SNPs (lower).

As shown in Figure 3a, the correlation between marbling score EPD and their fitted values in the training set (referred to hereafter as fitting accuracy) increased almost monotonically with panel size, until reaching a plateau at a panel size of 1400 SNPs. However, the correlation between marbling score EPD and their predicted values in the testing set (referred to hereafter as predictive accuracy) reached its peak (0.863) with a panel size of 700 SNPs, and decreased thereafter. The decrease in prediction accuracy with > 700 selected SNPs possibly reflects over-fitting in the training set, which, in this case, happened much before the panel size exceeded the training set size (i.e., approximately 2000 animals). Hence, with Bayesian regression models, prediction using more SNPs may not necessarily yield better results than prediction using a smaller panel, yet the optimal panel size may depend on many factors. In this study, we empirically chose the 700-SNP subset as the optimal panel. The fitting accuracy in the training set and predictive accuracy in the testing set using the optimal 700-SNP subset are illustrated in Figure 3b.

### Determination of an optimal ANN architecture

**Figure 4:**
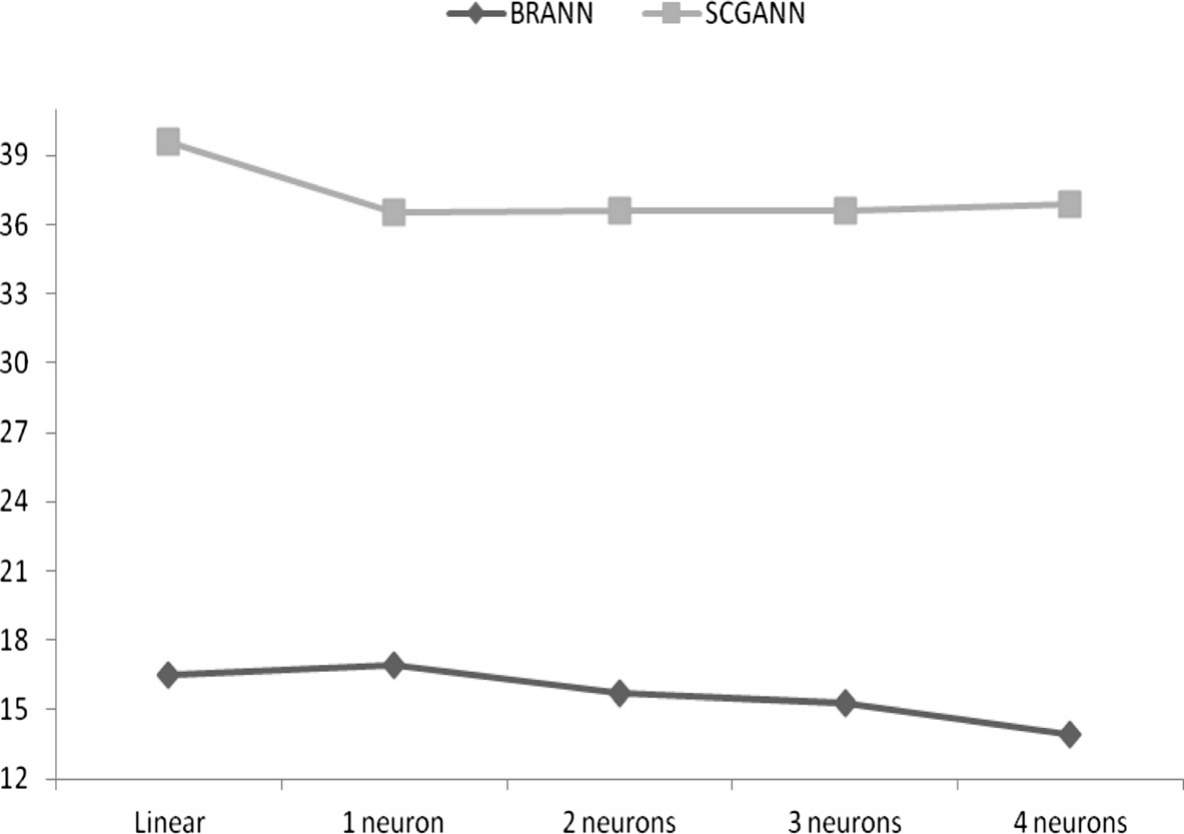
Sum of squared errors (y-axis) in the testing sets, computed as averages from eight replicates for Bayesian neural networks (BRANN) and ANN coupled with the SCG algorithm (SCGANN), for ANN with different numbers of neurons.

The performance of the ANN architectures was examined based on the sum of squared errors (SSE) of prediction with a 3K panel, averaged over eight independent replicates of each ANN, for both BRANN and SCGANN. Each ANN had a distinct combination of training algorithm, transformation method, and number of neurons, but both, BRANN and SCGANN, had an input of 3K SNPs. The average SSE ranged from 13.936 to 16.882 for BRANN, and from 36.531 to 39.571 for SCGANN. Smaller SSE were produced by BRANN with nonlinear activation functions and from 2 to 4 neurons, and by SCGANN with nonlinear activation functions and from 1 to 4 neurons. There was no evidence that more complex networks (e.g., with more neurons or a non-linear transformation function) produced better predictions than the linear model, as the ANN were similar in terms of their SSE. Possibly, this was because marbling score EPD is estimated under an additive model of inheritance in which additive genetic merit has a linear relationship with SNP effects. Nevertheless, BRANN performed as well as the linear models when predicting this linear system. Also, BRANN consistently produced a more accurate prediction of marbling score EPD than did SCGANN. On average, SSE obtained from BRANN were about 40% to 50% of those obtained from SCGANN (Figure 4). This is attributed to the use of Bayesian regularization, which handles over-fitting better than does the SCG back-propagation.

#### Predictive performance using the 3K-SNP panel

**Figure 5:**
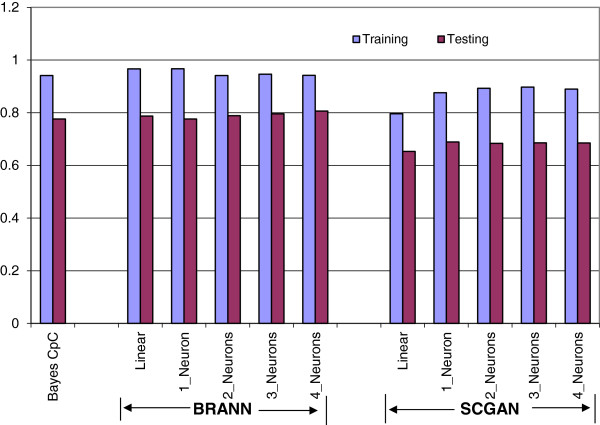
**Correlations between marbling score expected progeny differences in the training (testing) sets and their fitted (predicted) values using BayesCpC and BRANN and SCGANN with different numbers of neurons in the hidden layer and using the 3K-SNP panel. **^1^training = correlations in the training sets; testing = correlations in the testing sets; ^2^BayesCpC = Bayesian regression model, where the BayesCπ model is used for feature selection and BayesCπ with π = 0 is used for post-selection statistical inference and cross-validation; BRANN = artificial neural network with Bayesian regularization; SCGANN = artificial neural network with scaled conjugate gradient back-propagation.

BRANN and BayesCpC performed very similarly with the 3K-SNP panel and both methods yielded higher prediction accuracies than did SCGANN (Figure 5). On average, the correlation in the training set was 0.941 with BayesCpC. This correlation ranged from 0.942 to 0.967 with BRANN, and from 0.796 to 0.897 with SCGANN. The average correlation (in the testing set) was 0.776 with BayesCpC, and ranged from 0.776 to 0.807 with BRANN, and from 0.653 to 0.689 with SCGANN. In general, these correlations increased slightly with the number of neurons, but no consistent pattern was observed (Figure 5).

With the 3K-SNP panel, the number of SNPs (i.e., 2421) exceeded the number of animals (~2000 animals) in the training set. This means that there were more parameters to be estimated than data points, even when all SNPs entered the model linearly. This explains the better predictive performance of BRANN over SCGANN: Bayesian regularization imposes a penalty for model complexity and prevents over-fitting, while SCGANN captures both signal and noise in the training set, leading to poor prediction. The results illustrate that ANN with Bayesian regularization can behave better than ANN with SCG back-propagation. BRANN penalize the estimation of large weights to achieve a smoother mapping, which leads to effectively more parsimonious models.

**Figure 6:**
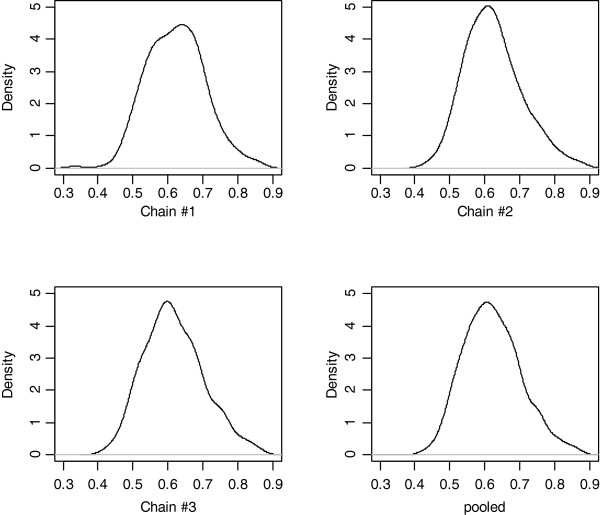
Posterior density for parameter π in the BayesCπ model obtained from three parallel chains and from pooled samples of the three chains.

In the BayesCpC procedure, the BayesCπ model postulates that a portion, π, of all SNPs have no effect on marbling EPD. In a high-density SNP panel, π is typically expected to be large, meaning that the portion of “signal” SNPs, 1 - π, is small, and the chance of over-fitting is reduced. Using the Illumina Bovine3K SNP genotypes, the posterior mean (standard deviation) of π was 0.621 (0.0830), based on 2421 polymorphic SNPs; this means that, on average, 918 SNPs were fitted in the model at each MCMC iteration. Hence, over-fitting was not a concern. Posterior densities for π are shown in Figure 6 and were computed using posterior samples obtained from each of the three parallel chains and from all pooled samples of the three chains. The density plots for the three parallel chains highly resembled each other and had similar means, indicating that convergence and length of the MCMC chains were appropriate for π. We noticed that the estimate of π obtained from the 3K panel was smaller than that based on BovineSNP50 genotypes, because the latter has typically been greater than 0.95 [5]. Thus, it would seem that, with a low-density SNP panel, the interpretation of SNPs having putatively non-zero effects on the target traits should be taken with caution, because many could be distant from the functional genes or quantitative trait loci. We also observed that the selected number of SNPs having non-zero effects based on BayesCπ in the training set did not correspond to the number of SNPs in the optimal SNP panel for prediction (918 vs 700 SNPs). We suspect that parameter π in a BayesCπ model does not fully inform on the size of an optimal SNP panel for prediction; a model that describes variation in a training set well does not necessarily yield the best predictions when generalized beyond the training set. This phenomenon is referred to as poor generalization in machine learning methods [12].

### Predictive performance using the700-SNP panel

**Figure 7:**
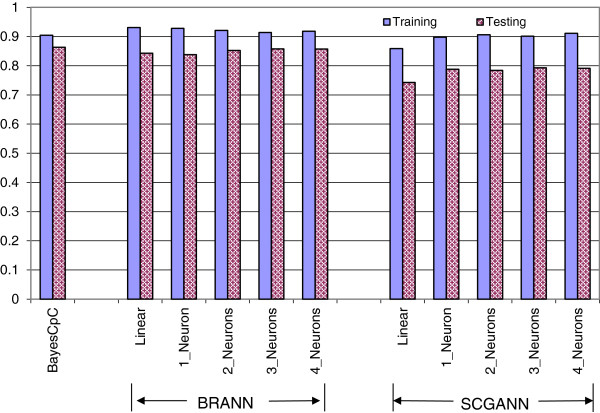
**Correlations between marbling score expected progeny differences in the training (testing) sets and their fitted (predicted) values using BayesCpC and BRANN and SCGANN with different numbers of neurons in the hidden layer and using 700 SNPs. **^1^training = correlations in the training sets; testing = correlations in the testing sets; ^2^BayesCpC = Bayesian regression model, where BayesCπ is used for feature selection and BayesCπ with π = 0 is used for post-selection statistical inference and cross-validation; BRANN = artificial neural network with Bayesian regularization; SCGANN = artificial neural network with scaled conjugate gradient back-propagation.

Unlike the case with 3K-SNPs, prediction using the selected 700-SNPs was not challenged by over-fitting. Hence, we observed a smaller difference in prediction performance between BRANN and SCGANN because regularization was not a decisive issue in the prediction. With the 700-SNP panel, the predictive performance of BayesCpC was slightly better than that of BRANN, possibly because the subset of SNPs was optimal, at least as selected by BayesCpC. As expected, BayesCπ and BRANN outperformed SCGANN in the prediction of marbling score EPD but differences in performance were smaller with the 700-SNP panel than with the 3K-SNP panel. Average prediction accuracy was 0.863 with BayesCpC and ranged from 0.843 to 0.858 with BRANN, whereas the average prediction accuracy with SCGANN ranged from 0.743 to 0.793. For both BRANN and SCGANN, the difference in predictive accuracy between the linear and non-linear activation functions was negligible (Figure 7).

## Discussion

We investigated the predictive performance of ANN in comparison to a hierarchical Bayesian regression model, using 3K-SNP and 700-SNP panels. The 700 SNPs were preselected from the former based upon their power to predict marbling EPD. Various ANN architectures to predict marbling score EPD were examined, in conjunction with two training algorithms, two types of activation functions, and from 1 to 4 neurons in the hidden layer. We did not observe significant differences in predictive accuracy between linear and non-linear models, probably because the relationship between marbling score EPD and SNP effects is theoretically linear. An EPD produces a smoothed data point based on additive inheritance, and this smoothing may mask non-linear variation in the response variable. A better way to analyze this trait would be to remove variation due to contemporary groups from the field data and then analyze individual marbling phenotypes, but this was not possible here because we did not have access to the raw data, which were, in general, collected on progeny of the genotyped bulls. The accuracy of EPD varies between individuals, which suggests that the residuals may be heteroscedastic due to unequal prediction error variances. An alternative is to use deregressed EPD as the target variable, for which the parent averages are removed and appropriate weights can be applied to account for heteroscedastic residuals [33]. Some reports suggest that training on deregressed EBV could generate higher accuracy of genome-enhanced breeding values than training on EBV [34]. However, the main purpose of the present research was to investigate the predictive performance of ANN in comparison with Bayesian regression models in a linear system. We used EPD instead of deregressed proofs because correct deregression involves matrix operations (the data available were incomplete for correct deregression), or approximations. Both EPD and deregressed EPD are heteroscedastic because of an unequal amount of information, but the neural network software used did not allow for incorporation of heteroscedasticity in a straightforward manner.

In genomic selection, the joint estimation of a genome-wide SNP set is challenging due to the so-called “large *p*, small *n*” problem, meaning that there are many more parameters to estimate than there are data points. This leads to over-fitting of the model in the training set and poor predictive performance when generalized to the testing set. With the 3K-panel, the number of animals (n ≈ 2000) in the training data set was less than the number of SNP markers (*p* = 2421). Hence, over-fitting might occur with linear regression models. The same is true for ANN models. In the ANN with 4 neurons, for example, there were approximately 9700 weights and bias parameters to estimate, which is much more than the number of data points in the training set. Hence, variance shrinkage (as occurs in the hierarchical Bayesian regression models) or Bayesian regularization (as occurs in BRANN) plays a crucial role in attenuating “over-fitting” and attaining reproducible predictive performance. In ANN, the effective number of parameters used in the model is typically less than the total number of weights, because some weights do not contribute due to shrinkage. Thus, over-fitting is attenuated and a model that generalizes well can potentially be attained [35]. In BRANN with the 3K-panel, for example, the effective number of parameters was 1282, 1213, 1031, 999 and 998, respectively, for linear and 1-, 2-, 3- and 4-neuron nonlinear architectures. These numbers are much smaller than the actual number of parameters in the models or the number of data points (ranging from 1946 to 2060 EPD) in the training set.

On the one hand, parametric statistical approaches have limited flexibility for modeling high-order non-linear interactions that may be important for complex traits [36,37]. On the other hand, neural networks have the potential to capture nonlinear relationships and may be useful in the study of quantitative traits under complex gene action, given suitable inputs. In a previous study, it was shown that non-linear neural networks outperformed a benchmark linear model when predicting phenotypes, especially in inbred wheat lines where cryptic gene interactions are expected [14]. In the present study, the predictive ability of BRANN was similar to that of BayesCpC with the 3K-panel and the selected 700-SNP panel. In addition, there was no difference in predictive ability between linear-ANN and non-linear ANN. We expected this because marbling score EPD are estimated under an additive linear model and so they should be predicted adequately under an ANN with a linear activation function. Nevertheless, we found that non-linear ANN with Bayesian regularization behaved as well as the linear models when predicting an additive target. Our results support the idea that ANN with Bayesian regularization can act as universal approximators of linear or non-linear functions of interest in breeding contexts.

Although BRANN consistently yielded better predictions than SCGANN, computing time with BRANN may restrict the application of these models. BRANN training updates the weights and biases using Levenberg-Marquardt optimization, and its computing time can increase drastically with the number of SNPs included in the model. For example, while it took only about 4 minutes to perform 1000 iterations for a BRANN with 1 neuron for the 700-SNP panel, 112 minutes were required for the 3K-SNP panel. The 3K-SNP analysis also consumed tens of times more memory. Thus, the application of BRANN to high-density chips (say 50K SNP or higher) is a significant challenge and improvements in the algorithms are needed before BRANN can be practically applied to genomic selection. One solution is to use (distributed) parallel computing, as we did with the high-throughput computing pipeline that implements hierarchical Bayesian regression models [38]. The SCG training algorithm was proposed to avoid the time-consuming search employed in BRANN, with significantly reduced computing time per iteration. However, the SCG back-propagation approach yielded worse predictions than both BRANN and the Bayesian regression models.

Finally, we found that feature selection may be important for Bayesian regression models because a model using all SNPs did not necessarily give the best prediction of marbling EPD. This situation is unlike ridge regression best linear unbiased prediction, or the G-BLUP method [38,39], where a model that includes all markers would typically be favored due to the increase in accuracy that comes with including additional markers.

## Conclusions

ANN with Bayesian regularization can perform as well as linear Bayesian regression models in predicting additive genetic values. ANN may be useful for predicting complex traits using high-dimensional genomic information and capture nonlinearities, and do so adaptively. While the selection of models of varying dimensions may be an issue worth exploring, it brings tremendous computing challenges, particularly when the data set is large. Hence, high-performance computing will be required for genomic selection using Bayesian regression models or artificial neural networks.

## Competing interests

The authors declare that they have no competing interests.

## Authors’ contributions

HO conceived and performed computations and drafted the manuscript; X-LW conceived, carried out the study, advised for computations, and revised the manuscript; RDS, JFT and BWW collected the samples and RDS and JFT generated the genotypes; GJMR, SB, BWW, RDS, JFT and DG helped conceive and coordinate the study, provided critical insights and revised the manuscript. All authors read and approved the final manuscript.
